# Influence of lincomycin-spectinomycin treatment on the outcome of *Enterococcus cecorum* infection and on the cecal microbiota in broilers

**DOI:** 10.1186/s13099-021-00467-9

**Published:** 2022-01-04

**Authors:** Jana Schreier, Daniela Karasova, Magdalena Crhanova, Ivan Rychlik, Silke Rautenschlein, Arne Jung

**Affiliations:** 1 Clinic for Poultry, University of Veterinary Medicine Hannover, Buenteweg 17, 30559 Hannover, Germany; 2 Veterinary Research Institute, Hudcova 296/70, 62100 Brno, Czech Republic

**Keywords:** *Enterococcus cecorum*, Infection, Cecal microbiota, Lincomycin, Spectinomycin, Broilers

## Abstract

**Background:**

*Enterococcus cecorum* (EC) is one of the main reasons for skeletal disease in meat type chickens. Intervention strategies are still rare and focus mainly on early antibiotic treatment of the disease, although there are no data available concerning the effectivity of this procedure. The present study aimed to investigate the effectivity of early lincomycin-spectinomycin treatment during the first week of life after EC-infection. Furthermore, the impact of lincomycin-spectinomycin treatment and EC infection on the development of cecal microbiota was investigated.

**Methods:**

A total of 383 day-old broiler chicks were randomly assigned to four groups (non-infected and non-treated, non-infected and treated, EC-infected and non-treated, and EC-infected and treated). The EC-infected groups were inoculated orally with an EC suspension at the day of arrival and at study day 3. The treatment groups were treated with lincomycin-spectinomycin via the drinking water for six consecutive days, starting two hours after the first inoculation. Necropsy of 20 chickens per group was performed at study days 7, 14, 21, and 42. Bacteriological examination via culture and real-time PCR was performed to detect EC in different extraintestinal organs. Cecal samples of nine chickens per group and necropsy day were analyzed to characterize the composition of the cecal microbiota.

**Results:**

No clinical signs or pathologic lesions were found at necropsy, and EC was not detected in extraintestinal organs of the EC-infected and treated birds. Lincomycin-spectinomycin promoted the growth of the bacterial genus *Escherichia/Shigella* and reduced the amount of potentially beneficial *Lactobacillus spp.* in the ceca regardless of EC-infection. Unexpectedly, the highest abundances of the genus Enterococcus were found directly after ending antibiotic treatment in both treatment groups, suggesting the growth of resistant enterococcal species. EC was not detected among the most abundant members of the genus *Enterococcus.* Oral EC-infection at the first day of life did not influence the development of cecal microbiota in the present study.

**Conclusions:**

Lincomycin-spectinomycin treatment during the first week of life can prevent the EC-associated disease in broiler type chickens and has a direct impact on the development of the cecal microbiota. The low abundance of EC in the ceca of infected chickens underlines the pathogenic nature of the disease-causing EC strains. Further research on alternative prevention and intervention strategies is needed with regard to current efforts on reducing the use of antibiotics in livestock animals.

**Supplementary Information:**

The online version contains supplementary material available at 10.1186/s13099-021-00467-9.

## Background

The bacterial species *Enterococcus cecorum* (EC) has become a major cause of disease outbreaks in the broiler industry worldwide [1–8]. The EC-associated disease, also called “enterococcal spondylitis” or “kinky back”, leads to an increase in mortality and therapy costs [2, 4, 7]. The typical course of the disease is characterized by a septic phase during the first three weeks of the production cycle, followed by the skeletal phase, which lasts from week three until the end of the cycle [9]. During the septic phase, affected birds can be asymptomatic or show non-specific symptoms such as depression, ruffled feathers, and retarded growth. Pericarditis and hepatitis are often found at this stage [2]. The first signs of lameness mark the onset of the skeletal phase of the disease. Affected birds suffer from progressive lameness and ataxia. Completely paralyzed birds are often seen in a typical sitting position and should be culled, as they are not able to reach feed or water anymore [4]. Necropsy reveals the main reason for paralysis: necrotic abscess material at the free thoracic vertebra constricting the spinal canal [7]. Another reason for lameness is femoral head osteomyelitis, which can lead to complete destruction of the cartilage and the underlying bone [10].

The fecal-oral transmission route is most likely in EC pathogenesis [11]. EC is known to be a commensal in the chicken’s intestine and was found in other birds and mammals as well [12]. Furthermore, it is thought to become the major enterococcal species in the gut of 12-week-old chickens [13]. Commensal strains start to colonize the gut in the third week of life. In contrast, pathogenic EC strains can be detected in ceca of chicks during the first week of life and may thus have a competitive advantage in colonizing the gut [9].

The role of the intestinal barrier function and the microbiota in enterococcal infections has not been the focus of studies on the EC infection until now [9]. Coinfection with other pathogens and immunosuppression have been thought to play a significant role in the EC pathogenesis [11, 14]. Unfortunately, no clear predisposing infectious factors could be identified so far. Different virulence factors known from other enterococcal species were found in some pathogenic EC strains from clinical cases, but their role in EC pathogenesis remains unclear [15–17]. To date, it is still unknown how EC translocates from the intestine to other tissues [14].

Early detection of the pathogen and diagnosis of the associated disease are important, as therapy has to start early to avoid increasing costs over time, and therapy of skeletal lesions in affected animals is not successful [18]. According to the literature, the drug of choice in treating the EC-associated disease is Amoxicillin [7, 8]. Enterococci are known to have a high level of antimicrobial resistance [19] and this has to be considered when treating the EC-infection. EC has been shown to be multi-drug resistant in several studies, and differences between commensal and pathogenic strains are usually found [16, 20–22]. Resistance to lincomycin was frequently detected in commensal isolates, whereas pathogenic isolates were often susceptible to this agent [16, 22]. Resistance to spectinomycin is even less frequent in EC isolates from ceca and spine lesions [20].

In the European Union (EU), lincomycin and spectinomycin are available as a combined soluble powder and approved for use in the drinking water in chickens and swine. The indication for use in chickens is the treatment of chronic respiratory diseases caused by *Mycoplasma gallisepticum* and *Escherichia coli*, and associated with low mortality rates [23].

The chicken gut microbiota is known to play an important role in health and disease. Extensive research has been conducted and reviewed intensively in recent years. Several studies showed that the cecal microbiota of healthy broilers comprises mainly three different phyla: *Firmicutes*, *Bacteroidetes*, and *Proteobacteria* [24–26]. Different factors, such as diet, age, and housing are thought to have a direct impact on the development and constitution of intestinal microbiota [26–29]. The chicken intestinal microbiota is most complex in the cecum and is thought to intensively interact within the microbial population itself as well as with the host’s immune system. The presence or absence of beneficial bacteria was linked directly to performance in meat type chickens [30]. As a result of high hygiene standards in modern broiler production, newly hatched chicks are relatively naïve regarding bacterial colonization of the intestine. Consequently, there is high variation in the composition of the cecal microbiota during the first two weeks of life. It is possible to manipulate the establishment of a stable bacterial community, and thus influence the health and productivity in the birds [31, 32]. There is no data available on the impact of early cecal EC colonization on the development of the intestinal microbiota. Antibiotic treatment is known to reduce the stability of microbiota in the gut and increase antimicrobial resistance [25].

Based on field observations, lincomycin-spectinomycin is used to treat EC-infections in Germany. Although extensive research on the microbial community in the chicken’s intestine has been conducted in recent years, there is no information on the effect of lincomycin-spectinomycin on the gut microbiota in broilers. Thus, the objective of this study was to investigate the impact of lincomycin-spectinomycin treatment on the EC infection and the gut microbiota in broilers.

## Results

### Clinical signs and pathology

In this experiment, symptoms of the EC-associated disease and typical gross lesions during the skeletal phase were only seen in the EC-infected, non-treated group (EN). The first non-specific symptoms were recorded at study day 13, and lameness was seen from study day 17 until the end of the study. In total, 36.9% of the birds in group EN showed non-specific symptoms including depression, ruffled feathers, and closed eyes, while 13.1% were found to be lame. Pericarditis was the most common gross lesion (26.2%), whereas hepatitis was found in 13.1% of the chickens. At study days 21–42, birds were also checked for spondylitis and femoral head osteomyelitis during regular and irregular necropsies. Spondylitis (15.9%) was found more often than femoral head osteomyelitis (4.5%). The non-infected groups (NN, NL) and the EC-infected group treated with lincomycin-spectinomycin (EL) did not show any EC-associated clinical signs or gross lesions throughout the trial. In group EN, four of 84 birds were euthanized due to animal welfare reasons. Necropsy was performed on all dead and euthanized birds according to the regular necropsy protocol. Three of these birds were EC-positive on culture, and spondylitis was found at necropsy. In group NN, two broilers were found dead in the first week (early chick mortality without any lesions), and in group NL, one broiler was euthanized due to ascites syndrome.

### Qualitative microbiology via culture and real-time PCR

**Fig. 1:**
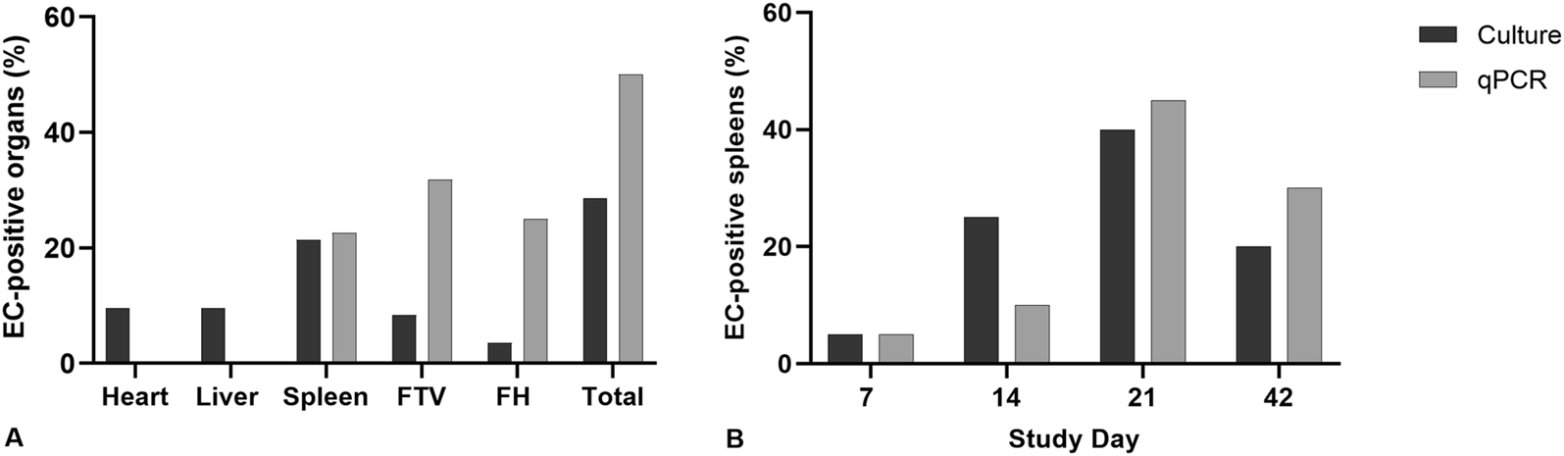
Microbiological examination via culture and real-time PCR in the EC-infected, non-treated group (EN). **A** EC-positive organs detected via culture and real-time PCR. All the hearts, livers and spleens were examined for EC via culture. FTV and FH were only examined on culture when gross lesions were detected at necropsy. Real-time PCR was performed for all the spleens at all necropsy days, and the free thoracic vertebra (FTV) and femoral heads (FH) at study days 21 and 42. Ct values below 36 were considered positive. **B** EC-positive spleens detected via culture and real-time PCR per study day. No significant differences in EC-detection were found between the two methods (*p* = 1.0, McNemar’s test) and results were substantially concordant (κ = 0.6416)

In total, 28.57% of the birds in the EN group were EC-positive on culture (Fig. 1 A). EC was mainly isolated from the spleen (21.43%), followed by the heart and liver (9.52% each). Isolation rates from these organs and the overall isolation rate were significantly higher compared to the other groups (*p* ≤ 0.05), as EC was not recovered from the birds in any other group. The number of EC-positive chickens in the EN group peaked at study day 21, with nine of 20 birds (45%) being EC-positive in either one or more of the examined organs (heart, liver, and spleen). The free thoracic vertebra (FTV) and the femoral heads (FH) were only bacteriologically examined when gross lesions were detected. Throughout the whole trial, 8.33% of the broilers in the EN group developed EC-positive gross lesions at the FTV. A smaller amount of birds (3.57%) had osteomyelitis lesions at the femoral heads that were positive for EC.

In real-time PCR, EC was detected in the spleen, the FTV, and the FHs of the EN group throughout the trial (Fig. 1 A). On comparing the results from the different organs at the time points where all the respective organs were sampled (study days 21 and 42), EC was detected mainly in the FTV (30%), followed by the spleen (22%), and the femoral heads (18%). In total, 50% (22/44) of the birds were EC-positive in either one or more of the examined organs. Spleen samples from study days 7 and 14 were additionally analyzed. The number of EC-positive spleens increased during the first three weeks and peaked at study day 21 (Fig. 1B). The Kappa coefficient and McNemar’s test were calculated for comparison of the detection rates of EC in the spleen via culture and real-time PCR. Results from culture and real-time PCR were substantially concordant (κ = 0.6416) and no significant difference between the two methods was found (*p* = 1.000). The number of EC-positive FTVs and FHs was highest at the end of the study. At study day 42, 45% of the FTVs and 25% of the FHs were EC-positive.

### EC detection in the cecum via quantitative real-time PCR

**Fig. 2:**
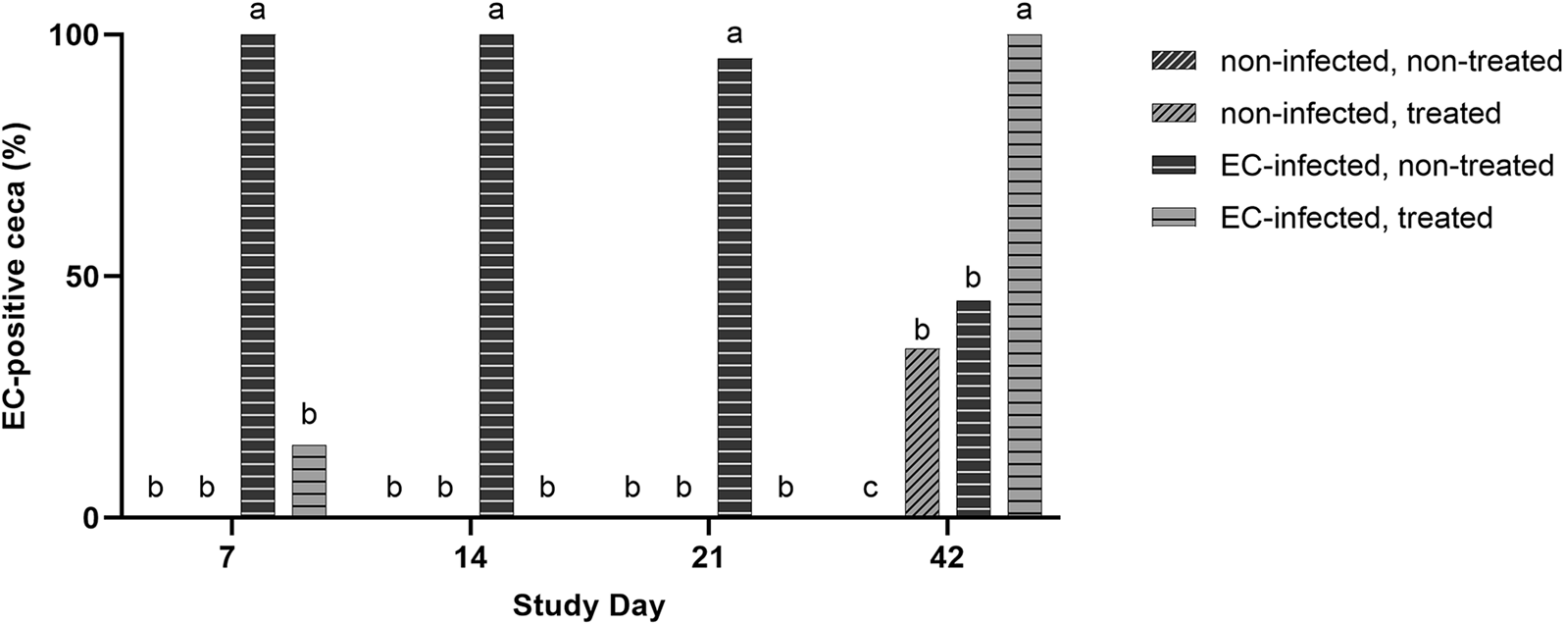
Cecal colonization by EC. Birds were infected with EC at study day 1, and treated groups received lincomycin-spectinomycin via the drinking water from study day 1 to 6. Samples were analyzed via real-time PCR and Ct values below 36 were considered positive. Different letters indicate significant differences between the groups per study day (*p* ≤ 0.05). Comparison between the groups was made for each study day by using Fisher‘s exact test. *p*-value adjustments for multiple testing were performed by using the Bonferroni-Holm correction method. N = 20 per group and study day

During the first three weeks, almost 100% of the tested broilers in the EC-infected, non-treated group (EN) were EC-positive in the cecum (Fig. 2). At study day 7, immediately after the end of antibiotic treatment, EC-DNA was detected in 20% of the birds in the EC-infected group with antibiotic treatment (EL). In this group, EC was not detected in the following two weeks (study days 14 and 21). However, at study day 42, EC was detected in all the birds from group EL. Furthermore, EC was found in approximately 45% of the birds in groups NL and EN at study day 42. The non-infected, non-treated group NN remained EC-negative throughout the study.

### Characterization of cecal microbiota

#### Sequencing coverage and depth

A total number of 154 cecal samples were analyzed. We obtained 7,214,356 reads, which led to an average coverage of 46,846 reads. The lowest coverage was 10,370 reads, the highest coverage 140,978 reads. These reads were distributed among 41,315 operational taxonomic units (OTUs).

#### Phylum level

**Fig. 3:**
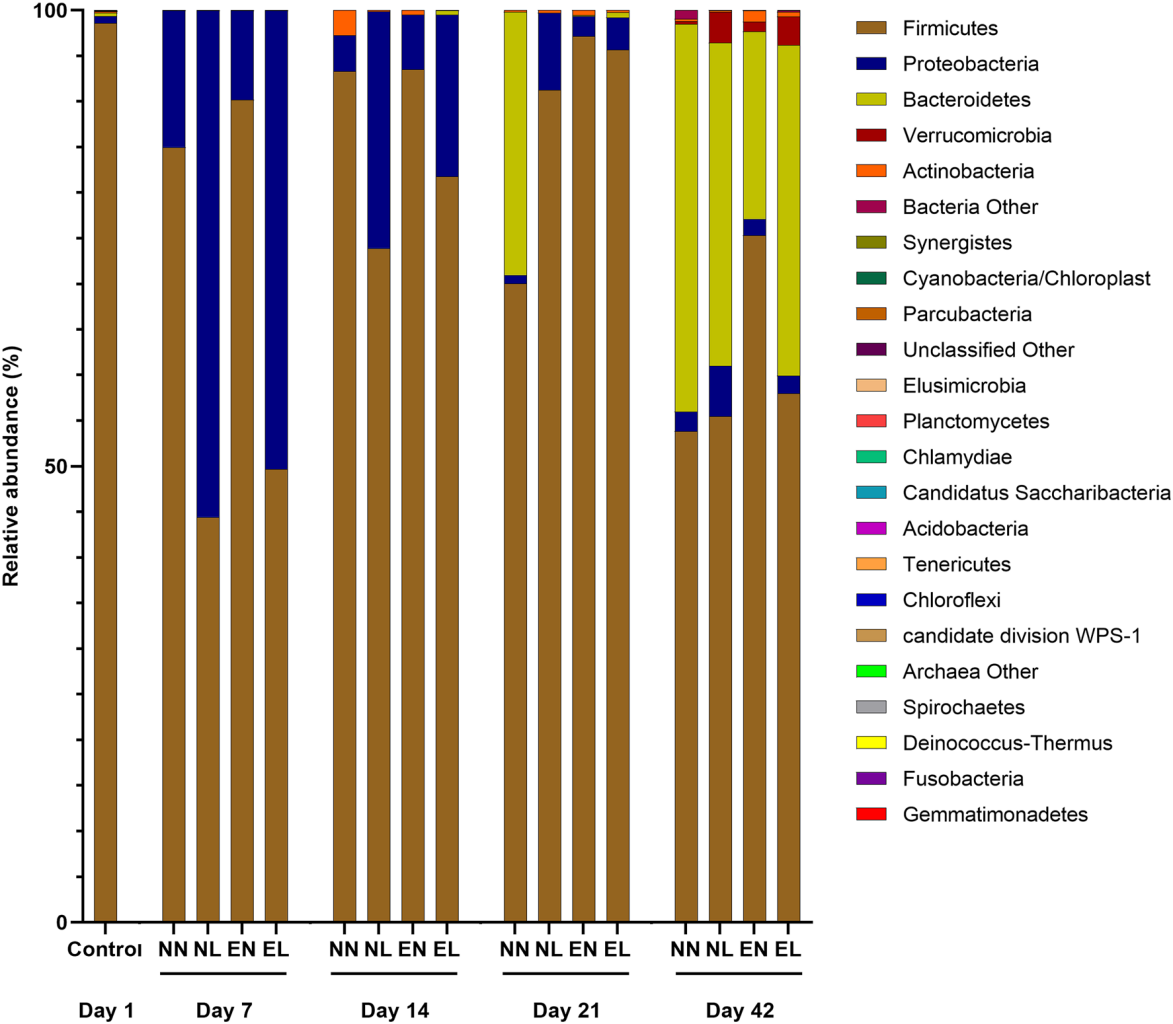
Characterization of cecal microbiota at phylum level. Data were analyzed using Qiime. Relative abundances (%) of each phylum are presented per study day and group. N = 9 per group and study day; *NN* non-infected, non-treated, *NL* non-infected, treated with lincomycin-spectinomycin, *EN* EC-infected, non-treated, *EL *EC-infected, treated with lincomycin-spectinomycin

In total, 22 different phyla were identified in this experiment. Independent of study day or group, the majority of these phyla (> 99%) was formed by *Firmicutes* (76.87%), *Proteobacteria* (12.45%), and *Bacteroidetes* (9.71%). The relative abundance of these phyla changed over time. On the first day of life, *Firmicutes* formed the majority of phyla identified in the ceca (98.56%), followed by *Proteobacteria* (0.75%), and *Bacteroidetes* (0.4%). At the first necropsy day, which was study day 7, and directly after the end of treatment with lincomycin-spectinomycin, clear differences between the treated and untreated groups were found. In groups NN and EN, *Firmicutes* represented the majority of phyla (84.95 and 90.17%, respectively), followed by *Proteobacteria* (15.02 and 9.79%, respectively). However, in groups NL and EL, *Proteobacteria* represented the majority of phyla (55.56 and 50.29%, respectively), followed by *Firmicutes* (44.4 and 49.66%, respectively). In the following weeks of the experiment, these differences became less clear and the relative abundance of *Firmicutes* increased in both groups treated with lincomycin-spectinomycin until study day 21 (> 90%). At study days 14 and 21, *Firmicutes* represented the predominant phylum in all four groups (> 70%). At study day 42, *Firmicutes* was still the phylum with the highest abundance in all four groups (> 50%), followed by *Bacteroidetes* (the relative abundance ranged between 20.53% (EN) and 42.45% (NN)) (Fig. 3).

#### Family and genus level

**Fig. 4:**
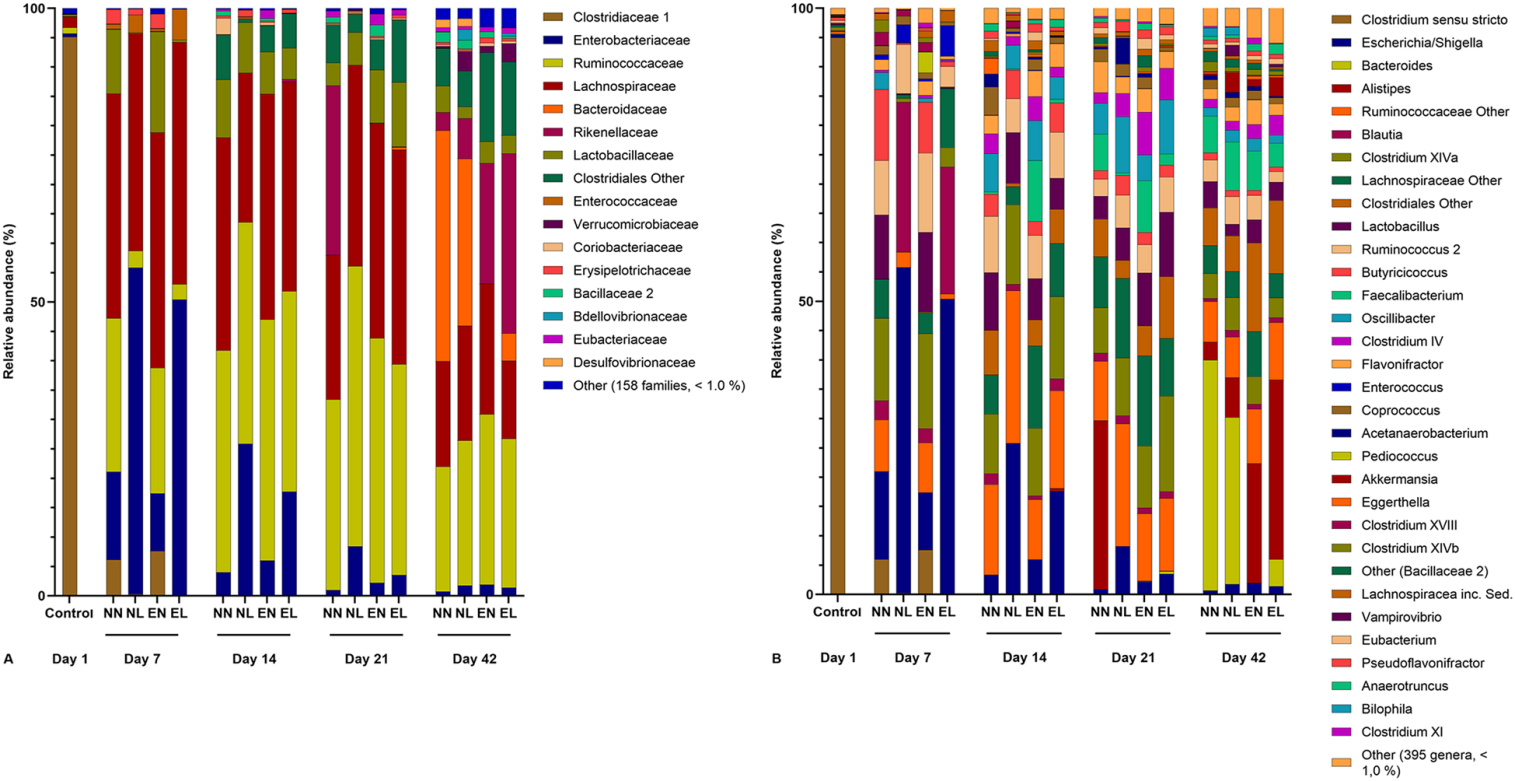
Characterization of cecal microbiota at family level **A** and genus level (**B**). Data were analyzed using Qiime. Relative abundances (%) of each family or genus are presented per study day and group. A total of 158 families and 395 genera were summarized as “Other” because the overall average of the relative abundance was below 1.0%. N = 9 per group and study day, *NN* non-infected, non-treated, *NL* non-infected, treated with lincomycin-spectinomycin; *EN* EC-infected, non-treated, *EL* EC-infected, treated with lincomycin-spectinomycin

To provide deeper insight into the microbial composition in the ceca of the different groups and the development over time, we performed further analysis at family and genus level. Data are shown in Fig. 4A (family level) and Fig. 4B (genus level). In total, 426 different genera from 173 bacterial families were identified in this experiment. At genus level, control birds at study day 1 (EC-negative status) were mainly colonized by the genus *Clostridium sensu stricto* (95%, family *Clostridiaceae 1*), followed by *Clostridium* XIVa (0.6%, family *Lachnospiraceae*, phylum *Firmicutes*) and *Escherichia/Shigella* (0.56%, family *Enterobacteriaceae*, phylum *Proteobacteria*). After the first week of life and right after the end of treatment at study day 7, the microbial composition had become more diverse in all four groups. In comparison to the first day of life, *Clostridium sensu* stricto was far less abundant in all four groups compared to the first study day, especially in the two treatment groups (6.0% in NN, 0.26% in NL, 7.59% in EN, and 0.16% in EL, respectively). In groups NN and EN, *Clostridium* XIVa (14.03% and 16.19%, respectively) and *Lactobacillus* (family *Lactobacillaceae*, phylum *Firmicutes*; 10.93% and 13.56%, respectively) were some of the most abundant genera. Both of these genera were far less abundant in the two treatment groups NL and EL (< 1%). In groups NL and EL, the most abundant genus was *Escherichia/Shigella* (> 50%), followed by *Blautia* (> 20%, family *Lachnospiraceae*). The genus *Escherichia/Shigella* was also found in the untreated groups NN and EN at study day 7, but at a relatively lower abundance (14.98% and 9.77%, respectively). One week later, at study day 14, the relative abundance of *Escherichia/Shigella* had decreased in groups NL and EL (25.79% and 17.68%, respectively). A further decrease towards the end of the study was seen as the microbial composition became more complex and various other genera were found in all four groups. In contrast to the first three necropsy days, the abundance of different genera in groups NN and NL was highly similar to each other as was the case in groups EN and EL at study day 42.

Alpha diversity indices and principal coordinate analysis for visualization of beta diversity further underlined differences seen at family and genus level. As expected, at the end of antibiotic treatment at study day 7, the untreated groups (NN, EN) were colonized by significantly more species than the lincomycin-spectinomycin treated groups (NL, EL; Table 1). Similar significant differences were found at that day for the Chao1 index, which estimates species richness, and the Shannon index, which estimates species diversity and takes into account species richness and evenness. At study days 14, 21, and 42, significant differences were found between groups for all three indices, but they were not the same for all three estimators.

**Table 1:** Alpha diversity in cecal samples

Study day	1	7				14			
Group	Control	NN	NL	EN	EL	NN	NL	EN	EL
Observed species	256 ± 74	606 ± 159^a^	315 ± 54^b^	732 ± 209^a^	351 ± 60^b^	1917 ± 462^a^	1262 ± 351^b^	3161 ± 919^a^	2264 ± 751^a^
Chao1 estimate	515 ± 114	1118 ± 296^a^	613 ± 92^b^	1425 ± 356^a^	732 ± 137^b^	4088 ± 978^ac^	2984 ± 921^a^	7427 ± 1948^b^	5356 ± 1642^bc^
Shannon index	0.59 ± 0.27	4.23 ± 0.38^a^	1.85 ± 0.17^b^	4.39 ± 0.45^a^	2.15 ± 0.68^b^	5.72 ± 0.19^a^	4.15 ± 0.39^b^	6.49 ± 0.44^c^	5.51 ± 0.61^a^

Study day		21				42			
Group		NN	NL	EN	EL	NN	NL	EN	EL
Observed species		2630 ± 880^a^	2075 ± 396^a^	2279 ± 836^a^	1358 ± 388^b^	1382 ± 275^a^	1458 ± 416^a^	2067 ± 568^a^	1801 ± 777^a^
Chao1 estimate		6923 ± 1831^a^	4524 ± 893^bc^	5438 ± 1609^ac^	3602 ± 800^b^	3971 ± 796^a^	3890 ± 1097^ab^	5867 ± 1308^b^	4840 ± 1810^ab^
Shannon index		5.77 ± 0.53^a^	5.84 ± 0.24^a^	6.35 ± 0.27^b^	6.10 ± 0.24^ab^	5.15 ± 0.36^a^	5.76 ± 0.33^b^	6.38 ± 0.31^c^	5.60 ± 0.73^ab^

Different superscript letters indicate significant differences between the groups per study day (*p* ≤ 0.05). Comparison between the groups was made by using the Kruskal-Wallis-Test and Mann-Whitney-*U*-Test, followed by the Bonferroni-Holm correction method for *p*-value adjustment for multiple testing. *NN* non-infected, non-treated, *NL* non-infected, treated with lincomycin-spectinomycin, *EN *EC-infected, non-treated, *EL* EC-infected, treated with lincomycin-spectinomycin

Composition of the gut microbiota in nine chickens per group and day was visualized by principal coordinate analysis (PCoA) based on weighted and unweighted UniFrac distance metric (Additional file 1). In general, chickens belonging to the same group shared highly similar microbiota at one time point and clustered closely together. The microbial cluster from one group at a specific time point was distinct from other groups at the same time point or the same group at another time point. At study day 7, the two treatment groups clustered close to each other but were distinct from the untreated groups which formed two other closely related clusters. In contrast, the two EC-infected groups (EN, EL) clustered separately from the non-infected groups (NN, NL) at study day 42. Furthermore, the samples from the two uninfected groups were relatively more distinct from each other within their respective cluster.

#### Genus *Enterococcus*

**Fig. 5:**
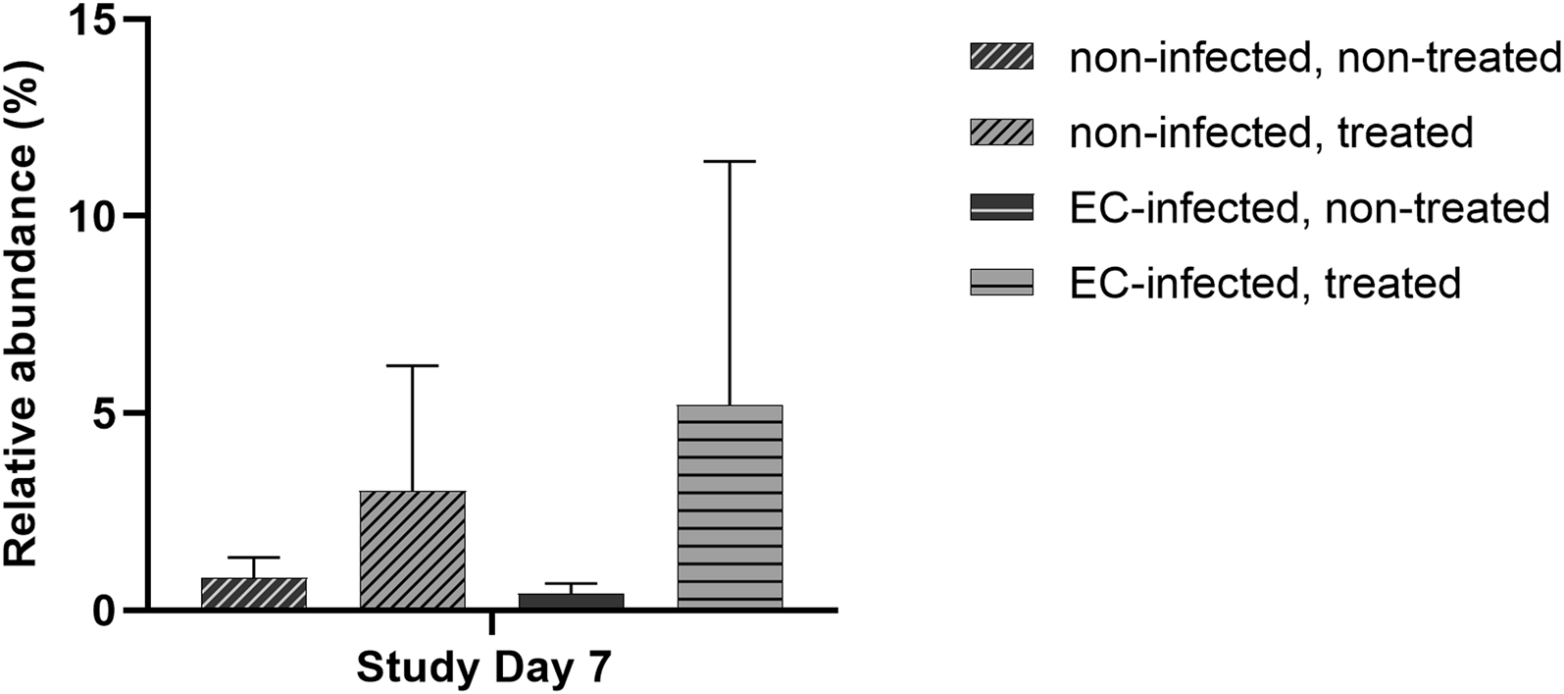
Relative abundances of the genus *Enterococcus* at study day 7 in the four different study groups. Bars represent the mean and standard deviation. *Treated* treated with lincomycin-spectinomycin

*Enterococcus* is a member of the family *Enterococcaceae* within the phylum *Firmicutes*. Among the 2300 most abundant OTUs in our study, we could find nine sequences assigned to the genus *Enterococcus*. Three of them were identified as *E. gallinarum*, two as *E. faecalis*, one as *E. faecium*, and three could not be defined exactly but were most likely to be *E. faecium*, *E. hirae* or *E. durans*. None of these sequences belonged to *Enterococcus cecorum.* A closer look at the relative abundance of the genus *Enterococcus* in the four groups at all the given time points revealed an unexpected outcome. In control birds at the first study day as well as in all groups at study days 14, 21, and 42, *Enterococcus* was lowly abundant in the cecal microbiota (< 0.05%). However, at study day 7, *Enterococcus* was the fourth most abundant genus in groups NL and EL (3.03 and 5.21%, respectively), but less abundant in groups NN and EN (0.83 and 0.42%, respectively; Fig. 5).

## Discussion

EC infections are one of the main problems in broiler production today. However, there is no data available concerning treatment of the disease. The aim of this study was to investigate an intervention strategy against EC-associated disease outbreaks. We examined the course of the EC-infection after inoculation on the first and third day of life, accompanied by an early onset of treatment with lincomycin-spectinomycin. In this experiment, the recommended dosage of lincomycin-spectinomycin (16.65 mg lincomycin and 33.35 mg / kg BW) for chickens was used in the drinking water for six consecutive days after the first inoculation. The strain EC14/086/4/A was sensitive to lincomycin and spectinomycin in antimicrobial susceptibility testing using disk diffusion tests. The antibiotic treatment successfully prevented the onset of the disease. None of the typical EC-associated clinical symptoms or gross lesions were found in the EC-infected, lincomycin-spectinomycin treated birds (EL). Furthermore, in this group, EC was not detected via culture or real-time PCR in any of the extraintestinal examined organs throughout the experiment. In contrast, clinical symptoms and pathologic lesions of the EC-associated disease were seen in the EC-infected, non-treated group (EN). The detection rates via culture and real-time PCR in this group correspond to morbidity rates reported from field outbreaks and experimental infection [2, 9, 14]. EC was frequently detected in the different examined extraintestinal organs and the typical course of the disease, including the septic and the skeletal phase, was successfully reproduced in this experimental group.

All the birds in group EN were colonized by EC throughout the first three weeks of the experiment. In contrast, EC-DNA was detected in 15% of the ceca in the EL group at study day 7, but not detected at all at study days 14 and 21, suggesting that no viable EC was left in this group after treatment. Interestingly, some of the birds of the respective group were colonized again at study day 42. We cannot exclude that the birds in group EL were colonized again by the experimental strain EC14/086/4/A, since it could have survived in the environment of the birds. On the other hand, it is possible that another unknown and probably commensal EC strain colonized the birds. This question remains unanswered, as we were not able to isolate the strain and thus could not characterize it further. While the non-infected, untreated group (NN) was not colonized by EC until the end of the study, 40% of the birds in the non-infected group treated with lincomycin-spectinomycin (NL) were colonized by EC at study day 42. It can be hypothesized that this group was colonized by a commensal EC strain, but the origin of this strain remains unclear. *Enterococci* are known to be widespread in the environment of broilers and can be found in litter and feed [33]. Recently, it has been shown that EC is highly durable and can survive on different materials and under various environmental conditions for quite a long time [34]. Even though our experiment was conducted under strict hygiene conditions, it is still possible that EC entered the stable via the feed, the litter or other biotic or abiotic resources [35], as any other gut microbiota member detected in the ceca of all the chickens used in this study did.

Interestingly, there were considerably low abundances of the genus *Enterococcus* among the cecal microbiota of all the study groups at all investigated time points. At study day 7, the relative abundance of the genus *Enterococcus* was higher in the two groups treated with lincomycin-spectinomycin compared to the untreated groups. Resistance against lincomycin-spectinomycin of different enterococcal species from poultry was investigated in 2016 [36]. It was shown that the majority of all enterococcal isolates were resistant against lincomycin-spectinomycin. We therefore suggest that the antibiotic treatment suppressed the growth of major microbiota members but promoted the growth of other lincomycin-spectinomycin resistant *Enterococcus* species. Additionally, EC has been shown to be one of the most predominant enterococcal species in poultry, but less frequently isolated than *E. faecalis* and *E. faecium* [36]. This might explain the fact that we were not able to find sequences of EC among the top 2300 OTUs in the present study. In general, interaction within the cecal microbiota as well as between the microbes and the host is highly complex. Furthermore, specific bacteria associated with disease in chickens and humans (e.g. *Clostridium difficile* [37], *Campylobacter spp.* [29], and *Salmonella enterica* serovar Enteritidis [38]), have been studied in regard to their influence on the microbiota composition with divergent outcomes. Results from these studies led to the conclusion that not necessarily a single species but rather a combination of different bacterial groups and their complex interactions are of vital importance for the development of the cecal microbiota [37]. The low abundance of the genus *Enterococcus* in our study suggest that it is not necessary for EC to be a predominant species in the cecum to be able to cause the disease. This seems to be an important finding and underlines the pathogenic nature of disease-causing EC strains. Similar data have been found for *Escherichia coli (E.coli)*, which is less abundant in the ceca of healthy chickens [39, 40]. Nevertheless, pathogenic strains of *E. coli* are the causing agent of colibacillosis, the most common infectious bacterial disease in poultry [41].

EC-infection did not influence the development of the cecal microbiota and the species diversity in our study, as the composition of the cecal microbiota as well as alpha and beta diversity in the two untreated groups NN and EN was highly similar within the first two weeks of the experiment. Differences at later time points cannot be explained only based on the two influencing factors antibiotic treatment and EC-infection, but rather by several environmental factors, including litter and feed. It is most likely that the cecal microbiota within each physically separated experimental group developed individually until the end of the study. The development of cecal microbiota in chickens has been studied intensively in the past. Initial exposure of naïve chicks to different bacterial communities can lead to distinct microbiota [32], and differences in microbiota diversity depend on many factors such as age [37], diet [30], and litter [27]. Thus, finding possible intervention strategies to prevent bacterial diseases, such as prebiotics, probiotics or competitive exclusion, is very challenging [42]. Knowledge about the composition and development of cecal microbiota in young broiler chickens may be important for the development of preventive methods against the EC infection.

In addition to data on cecal colonization under EC-infection, the present study provides an overview on the development of cecal microbiota under lincomycin-spectinomycin treatment. Usage of antimicrobials is known to disturb the homoeostasis of the gut microbiota [25]. Accordingly, we wanted to further characterize the effect of treatment on the development of the cecal microbiota throughout the experiment. We could show that antibiotic treatment has a direct impact on the composition of the cecal microbiota. Development of the cecal microbiota in untreated groups resembled the development of cecal microbiota in healthy broilers with *Firmicutes, Proteobacteria*, and *Bacteroidetes*, being the most abundant phyla in all experimental groups at all given time points [24, 25]. The treatment groups NL and EL were colonized by members of the genus *Escherichia/Shigella* in high abundance after treatment. This leads to the suggestion that treatment with lincomycin-spectinomycin could enable colonization by other facultative pathogens such as multidrug-resistant avian pathogenic *E. coli*. Lincomycin is not active against *E. coli* [43]. Resistance against spectinomycin is widespread among avian *E. coli* isolates and often associated with multi-drug resistance [44, 45]. Nevertheless, lincomycin-spectinomycin is approved for treatment of *E. coli* infections in poultry. However, antibiotic treatment of enterococcal infections with lincomycin-spectinomycin may lead to the selection of pathogenic *E. coli* and should be administered cautiously. Additionally, members of the genus *Lactobacillus* were frequently found in the untreated groups NN and EN, but far less so in the lincomycin-spectinomycin treated groups NL and EL. *Lactobacilli* are described as beneficial bacteria in the literature and often used in pro- and synbiotics [46]. Several studies have shown that *Lactobacillus spp.* can decrease colonization of different bacteria, including *Campylobacter jejuni* [47], *Salmonella enterica* [48], and *E. coli* [49]. This leads to the suggestion that treatment with lincomycin-spectinomycin could not only enable pathogenic bacteria to excessively colonize the gut. Treatment also seems to reduce the amount of beneficial bacteria in the gut which could otherwise control potentially harmful bacteria.

Our results show that the early onset of treatment with lincomycin-spectinomycin can prevent the EC-associated disease in broiler chickens. In general, antibiotic resistance to lincomycin is relatively high, whereas resistance to spectinomycin and other aminoglycosides tends to be rather low in EC [50]. However, the antibiotic resistance profile of the respective pathogenic EC strain has to be taken into account when treating field outbreaks. The success of the treatment should be verified continuously in order to be able to react in time if treatment fails, as chronic bone lesion treatment in affected birds is impossible [7, 8]. In regard to future perspectives and current efforts on reducing the use of antibiotics in livestock animals, other preventive methods are needed, which include identifying transmission routes, effective cleaning and disinfection, vaccination strategies, and potential use of probiotics. Further research is required to find suitable preventive intervention strategies.

## Conclusions

In conclusion, treatment of the EC infection with lincomycin-spectinomycin successfully prevented the onset of the EC-associated disease in our study. In addition, this study provides first insights into the development of cecal microbiota during EC infection. Whereas antibiotic treatment had a direct impact on the composition of the cecal microbiota, surprisingly, no clear differences were found between the EC-infected and non-infected groups. Pathogenic EC strains seem to play a minor role within the microbial community in the ceca of broiler chickens, but nevertheless lead to severe disease outbreaks. Further research is needed to understand the role of the intestinal microbiota in EC pathogenesis and to find alternative preventive intervention strategies.

## Materials and methods

### Animals and housing

A total of 383 one-day-old broiler chicks (Ross 308, obtained from Brüterei Weser-Ems GmbH & Co. KG, Visbek, Germany) were housed in floor pens on wood shavings in isolation units at the Clinic for Poultry, University of Veterinary Medicine Hannover, Foundation, Hannover, Germany. Upon arrival, 10 birds were submitted to necropsy and the remaining chicks were randomly divided into four groups. A broiler standard diet (Deuka, Deutsche Tiernahrung Cremer GmbH & Co. KG, Duesseldorf, Germany) was fed *ad libitum* throughout the trial. Starter feed was provided from days 1 to 10, followed by grower diet from days 11 to 35, and finisher diet from day 36 to 42 (Table 2). The light program was 24 h of light on the day of placement, followed by 15 h of light from 07:30 to 22:30 until the end of the study, and standard temperature conditions were adjusted throughout the experiment [51].

**Table 2:** Composition of starter, grower, and finisher diet

Ingredients	Starter diet (Days 1–10)	Grower diet (Days 11–35)	Finisher diet (Days 36–42)
Crude protein (%)	21.50	20.00	18.00
Crude fat (%)	4.90	5.00	2.80
Crude cellulose (%)	3.90	3.30	3.00
Crude ash (%)	5.40	4.90	5.40
Lysine (%)	1.25	1.05	0.80
Methionine (%)	0.55	0.50	0.40
Calcium (%)	0.90	0.80	1.00
Phosphorus (%)	0.60	0.50	0.60
Sodium (%)	0.14	0.14	0.15
Metabolizable energy (MJ ME/kg)	12.40	12.40	12.00

Data are summarized based on the manufacturer’s indications (Deuka, Deutsche Tiernahrung Cremer GmbH & Co. KG, Duesseldorf, Germany).

### Experimental set-up

On the day of arrival, 10 birds were euthanized and checked for their EC-negative status (yolk sac, cecum). The remaining 373 birds were randomly divided into four groups and inoculation was performed. The first group was a non-infected, non-treated negative control group (NN). The second group was a non-infected group treated with Lincospectin® (222 mg/g lincomycin and 444.7 mg/g spectinomycin, Zoetis Deutschland GmbH, Berlin, Germany) via the drinking water (NL). Birds in the third group were infected with EC14 but not treated with the antibiotics (EN). The fourth group was infected with EC14 and treated with Lincospectin® (EL). Birds were inoculated orally with 0.5 mL of an EC suspension at study day 1 directly after arrival, and again at study day 3. Unintentionally, for the first inoculation, the bacterial concentration in the inoculum was too low (10^4^ colony forming units (CFU) in 0.5 mL), so a second inoculation was performed at study day 3. On that day, birds were infected orally with 0.5 mL of an EC suspension containing 10^8^ CFU. After inoculation, chicks had no access to water for 2 h. Birds in the treatment groups were treated with Lincospectin® via the drinking water for 4 days after the second inoculation until study day 6. The dosage was 2.5 g Lincospectin® powder per 10 L water (= 75 mg / kg body weight as recommended by the manufacturer). A daily monitoring of clinical signs, including depression, ruffled feathers, closed eyes (non-specific symptoms), and lameness, was performed in all four groups throughout the trial. Severely affected birds were euthanized and submitted to necropsy. Regular necropsies of 20 broilers per group were performed at study days 7, 14, 21, and 42. Gross lesions were documented at necropsy. The heart, liver, and spleen of all the birds were sampled for microbiological examination using Amies medium swabs (Hain Lifesciences GmbH, Nehren, Germany). Furthermore, dry swabs (Applimed SA, Châtel-St-Denis, Switzerland) from the spleen and the cecum were taken from all the birds for real-time PCR, and cecal samples were taken from nine chickens per group for the analysis of cecal microbiota. At study days 21 and 42, the free thoracic vertebra and the femoral heads were examined for pathologic lesions. Briefly, the respective sampling sites were exposed and cut sagittally in order to assess gross lesions at the cartilage and the underlying bone. Subsequently, dry swabs were taken from the bone marrow for real-time PCR and stored at – 20 °C for at maximum six weeks until further analysis.

### Challenge isolates and preparation of the inoculate

The EC isolate EC 14/086/4/A was used for challenge. This pathogenic strain was isolated from an EC-associated disease outbreak in a commercial broiler flock in 2014 and further characterized in a previous study [17]. The bacterial strain was thawed and grown on Columbia sheep blood agar (Oxoid GmbH, Wesel, Germany) at 37 °C for 20 h under microaerophilic conditions. Subcultures were prepared on the following day and incubated for another 20 h before preparing the inoculum. Colony material was dissolved in physiological saline solution at room temperature up to an optical density of 1.1 McFarland (McF; DENSIMAT; BioMérieux, Nuertingen, Germany), which corresponds to an EC concentration of 2 * 10^8^ colony-forming units per milliliter (CFU/mL). This initial solution was diluted 1:100 to achieve a final concentration of 2 * 10^6^ CFU/mL, and the inoculum was stored at room temperature until challenging the birds. Parallel to inoculation, the total bacterial count was determined to confirm the actual concentration of the inoculum. Due to technical problems with determining the optical density, animals were inoculated with 2 * 10^4^ CFU/mL at the day of placement and again with a second inoculum containing 2 * 10^8^ CFU/mL at day three. Antimicrobial susceptibility testing of our strain was performed prior to the trial. EC14 was sensitive to lincomycin and spectinomycin using the disk diffusion test (Oxoid GmbH).

### Qualitative microbiology via culture

EC-isolation was performed on Columbia colistin–nalidixic acid (CNA) agar (Oxoid GmbH) from Amies medium swabs taken at necropsy. The plates were incubated for 24 h at 37 °C and then screened for colonies of EC (small, gray, mucoid colonies with slight alpha-hemolysis). Pure subcultures from respective colonies were produced on Columbia sheep blood agar. After another 24 h of incubation, catalase and oxidase testing were performed and Gram staining of colony material complemented the microbiological examination. Besides typical colony morphology, isolates were identified as EC when they were oxidase and catalase negative, and gram positive to gram variable ovoid cocci were seen under the light microscope. Bacterial isolates that were not reliably identified by these methods were further analyzed via 16 S rRNA partial gene sequencing at Microsynth AG, Lindau, Germany [52–54].

### DNA isolation and quantitative real-time PCR

DNA isolation was performed from dry swabs using a commercial isolation kit (InnuPrep DNA Mini Kit, Analytik Jena AG, Jena, Germany) in accordance with the manufacturer’s instructions with minor modification. Only 30 µL of the elution buffer were used for the last step of the procedure instead of the recommended 200 µL. After determining the total DNA amount using the NanoDrop® ND-1000 Spectrophotometer (Thermo Fisher Scientific Inc., Wilmington, NC, USA), DNA was stored at − 20 °C until further use.

Each sample was analyzed in duplicate using a modified set-up of a recently published real-time PCR assay [55]. Each run was performed on 96-well-plates (Applied Biosystems™, Fisher Scientific GmbH, Schwerte, Germany) using the QuantStudio 3 Real-Time-PCR-System (Thermo Fisher Scientific Inc.). The same primers, probes, and polymerase were used as described in the respective article [55], but only half of the volume of all ingredients was used per well. Initial denaturation was set at 95 °C for 10 min, followed by 40 amplification cycles at 95 °C for 15 s, and 60 °C for 60 s. Mean Ct values above 36 were considered negative during data analysis.

### Characterization of cecal microbiota

Sequencing of the V3-V4 hypervariable region of 16 S rRNA genes from nine cecal samples per group and day was performed as previously described [56]. Sequencing results were analyzed and classified with RDP Seqmatch using the Qiime software [57]. The OTU (operational taxonomic unit) discrimination level was set to 97%. As we were interested in the enterococcal species found in the analysis of the cecal microbiota, we tried to identify the most abundant OTUs that were assigned to the genus Enterococcus by using the NCBI Basic Local Alignment Search Tool (BLAST).

### Statistical analysis

Data were statistically analyzed with SAS Enterprise Guide (Version 7.15, SAS Institute Inc., Cary, NC, USA) and graphs were created using GraphPad Prism (Version 9.2, GraphPad Software, LLC, San Diego, CA, USA). A descriptive statistical analysis was performed for clinical signs, pathology, and the composition of cecal microbiota at different taxonomic levels. Results from bacteriological examination via culture and real-time PCR were compared between groups using the Fisher’s Exact Test. Results from culture and real-time PCR within the same group were compared using the Kappa coefficient [58] and McNemar’s Test. Differences were considered significant at *p* ≤ 0.05. In order to determine within sample diversity (alpha diversity), the diversity estimators Observed species, Chao1 index, and Shannon index were calculated in Qiime [57]. Differences in alpha diversity between groups were analyzed using the Kruskal-Wallis test and Mann-Whitney *U* test, as conditions of normality and heterogeneity of variance were not met. The Bonferroni-Holm correction method for multiple testing was used to adjust *p*-values where applicable [59]. Principal coordinate analysis (PCoA) based on the weighted UniFrac analysis implemented in Qiime was used to visualize beta diversity.

## Supplementary Information

**Additional file 1. **Beta diversity of cecal microbiota visualized in principle coordinate analysis based on weighted UniFrac distance metric implemented in Qiime. Dots of the same color represent nine samples from one of the four study groups at one of the sampling days. *NN* non-infected, non-treated, *NL* non-infected, treated with lincomycin-spectinomycin, *EN* EC-infected, non-treated, *EL *EC-infected, treated with lincomycin-spectinomycin

## Data Availability

All data generated or analyzed during this study are included in this published article and its supplementary information files.
