# Supplementary figures and images for: Influence of lincomycin-spectinomycin treatment on the outcome of Enterococcus cecorum infection and on the cecal microbiota in broilers
Source: Gut Pathog. 2022 Jan 4;14:3. doi: 10.1186/s13099-021-00467-9 (PMC8729143; doi:10.1186/s13099-021-00467-9)

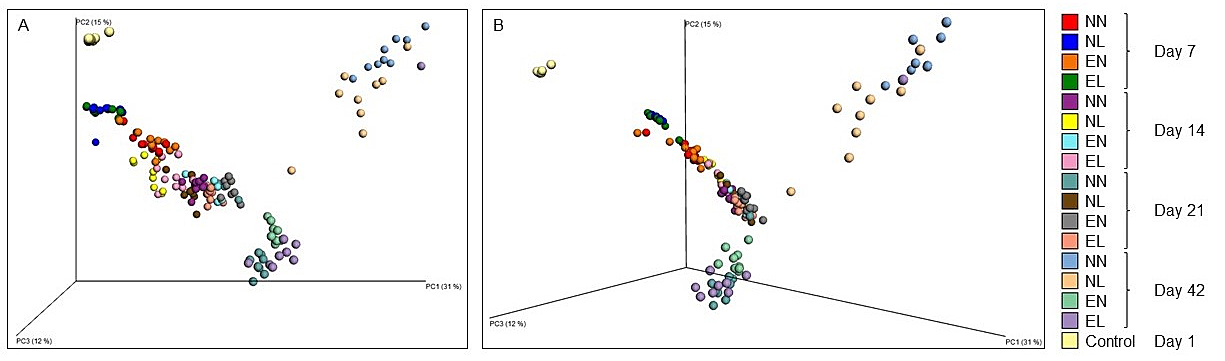

Supplement: Supplementary file 1 — Additional file 1. Beta diversity of cecal microbiota visualized in principle coordinate analysis based on weighted UniFrac distance metric implemented in Qiime. Dots of the same color represent nine samples from one of the four study groups at one of the sampling days. NN non-infected, non-treated, NL non-infected, treated with lincomycin-spectinomycin, EN EC-infected, non-treated, EL EC-infected, treated with lincomycin-spectinomycin [file 13099_2021_467_MOESM1_ESM.tif]
